# No Association Between Thrombus Perviousness and Cardioembolic Stroke Etiology in Basilar Artery Occlusion Stroke

**DOI:** 10.3389/fneur.2021.712449

**Published:** 2021-09-09

**Authors:** Anna Kufner, Matthias Endres, Michael Scheel, Christoph Leithner, Christian H. Nolte, Ludwig Schlemm

**Affiliations:** ^1^Klinik und Hochschulambulanz für Neurologie, Charité—Universitätsmedizin Berlin, Corporate Member of Freie Universität Berlin, Humboldt-Universität zu Berlin, Berlin Institute of Health, Berlin, Germany; ^2^Center for Stroke Research Berlin, Charité—Universitätsmedizin Berlin, Berlin, Germany; ^3^Berlin Institute of Health, Berlin, Germany; ^4^German Center for Cardiovascular Research, Partner Site Berlin, Berlin, Germany; ^5^German Center for Neurodegenerative Diseases, Partner Site Berlin, Berlin, Germany; ^6^Department of Neuroradiology, Charité—Universitätsmedizin Berlin, Corporate Member of Freie Universität Berlin, Humboldt-Universität zu Berlin, Berlin Institute of Health, Berlin, Germany

**Keywords:** basilar artery occlusion, ischemic stroke, computed tomography, thrombus, perviousness

## Abstract

**Background:** Thrombus perviousness (TP) quantified by thrombus attenuation increase (TAI) assessed on acute non-contrast computed tomography (NCCT) and CT angiography (CTA) may be associated with stroke etiology in anterior circulation ischemic stroke. We investigated whether TP is associated with stroke etiology and recanalization after mechanical thrombectomy in patients with acute basilar artery occlusion (BAO).

**Methods:** Eighty patients with complete BAO and in-house acute imaging from a prospectively maintained database were included. Two raters independently segmented the complete thrombus on co-registered NCCT and CTA to determine TAI in Hounsfield units (HU_CTA_–HU_NCCT_); averaged values of the raters were used for analysis. Recanalization to modified treatment in cerebral ischemia (mTICI) score 2b/3 was considered successful, and 90-day modified Rankin Scale score 0–2 was considered favorable.

**Results:** TAI did not differ between patients with different stroke etiologies; median TAI in patients with cardioembolic stroke (*n* = 36) was −0.47 (interquartile range −4.08 to 7.72), 1.94 (−8.14 to 10.75) in patients with large artery atherosclerosis (LAA; *n* = 25), and −0.99 (−6.49 to 5.40) in patients with stroke of undetermined origin (*n* = 17; *p* = 0.955). Binary logistic regression analyses did not identify TAI as an independent indicator of cardioembolic stroke (adjusted odds ratio [OR] vs. LAA stroke: 1.0 [95% CI: 0.95–1.0], *p* = 0.751). There was no association with successful recanalization (adjusted OR 1.4 [0.70–2.7], *p* = 0.345) or favorable outcome (adjusted OR 1.1 [95% CI: 0.94–1.2], *p* = 0.304).

**Conclusion:** In contrast to proximal middle cerebral artery occlusions, TP in BAO patients is not associated with cardioembolic stroke etiology. Larger confirmatory studies to establish the potential role of TP for clinical applications should focus on patients with anterior circulation stroke.

## Introduction

Basilar artery (BA) occlusion (BAO) is less frequent than anterior circulation stroke and occurs in approximately 10% of all patients with large vessel occlusions (1, 2). While outcome following BAO has been generally poor in the past (3), with the availability of endovascular therapies (EVTs), up to one-third of BAO patients achieve functional independence (modified Rankin Scale [mRS] score 0–2) (2, 4, 5). Similar to anterior circulation stroke, accurate etiological classification and identification of a cardioembolic source are essential to guide long-term secondary prevention in patients with BAO. However, despite its significance for therapeutic decision making, no cause can be identified after diagnostic workup in 22–35% of patients with BAO (2).

In patients with acute stroke and occlusion of the proximal M1 segment of the middle cerebral artery (MCA), thrombus perviousness (TP)—quantified by thrombus attenuation increase (TAI) on computed tomography (CT) angiography (CTA) relative to non-contrast CT (NCCT)—is associated with cardioembolic stroke etiology (6, 7). In the current study, we analyzed whether TP on admission CT is also associated with cardioembolic stroke etiology, recanalization rates, and functional outcome following EVT in patients with BAO.

## Methods

The raw data are not publicly available, as they contain information that could compromise patient privacy; anonymized summary data can be provided by the corresponding author upon reasonable request.

We performed an investigator-initiated retrospective study including patients from three different sites of our university hospital. Acute ischemic stroke with BAO enrolled in our prospectively maintained in-house database of consecutive patients undergoing EVT between 2015 and 2020 were eligible for inclusion. Inclusion criteria were as follows: NCCT and CTA on admission in a single session, showing complete BAO, and no administration of intravenous thrombolysis before imaging. Clinical data including demographic baseline data, presumed stroke etiology according to the trial of ORG 10172 in acute stroke treatment (TOAST; determined by local investigators unaware of TP who treated patients on certified stroke units according to current guidelines), and recanalization status were extracted from the database. Recanalization to modified treatment in cerebral ischemia (mTICI) score ≤ 2b/3 was considered successful, and 90-day mRS score 0–2 was considered favorable.

**Figure 1:**
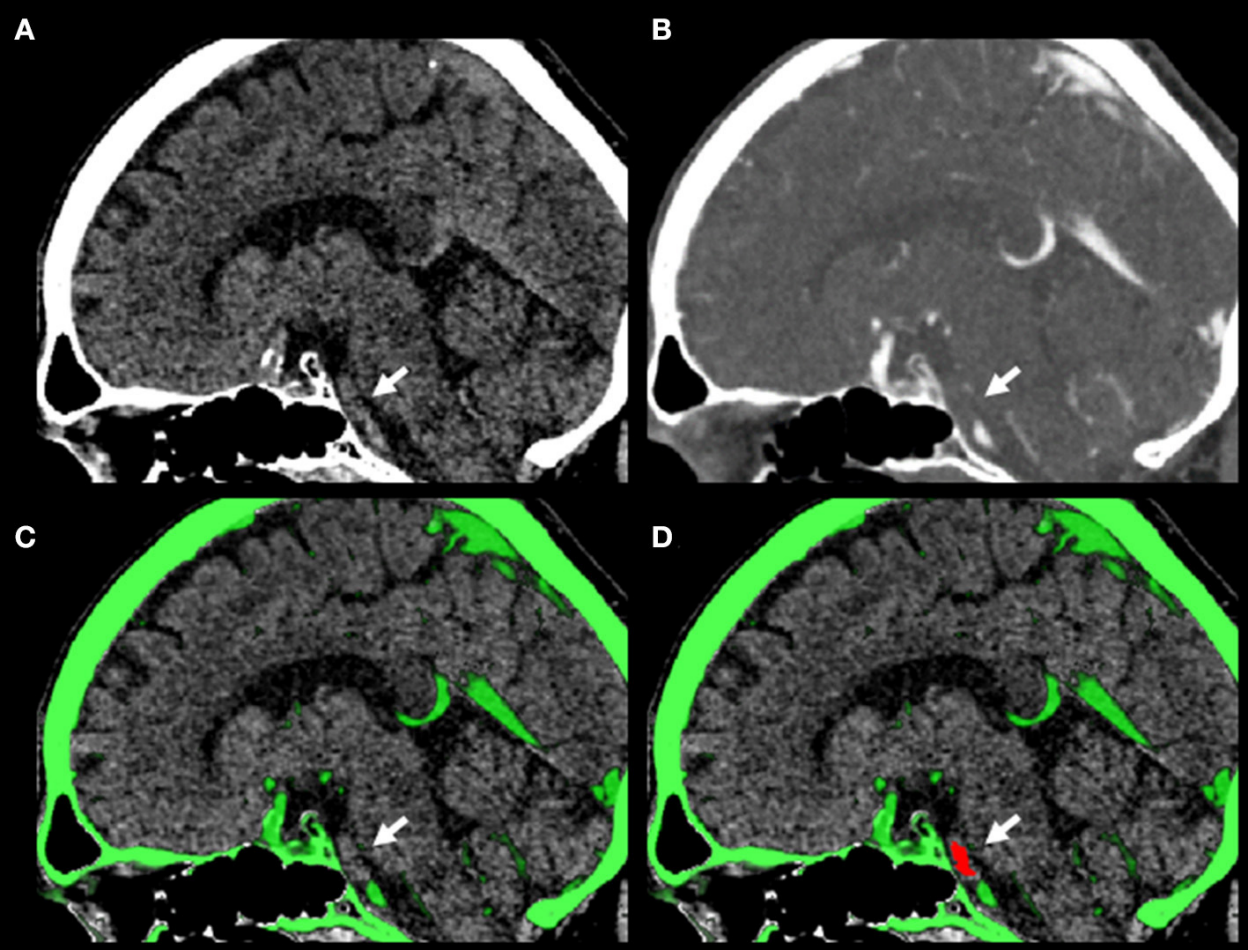
Example of a 66-year-old male patient presenting with a National Institutes of Health Stroke Scale (NIHSS) score of 20. No intravenous thrombolysis was performed due to arrival beyond 4.5 h since symptom onset. Mean thrombus attenuation increase (TAI) was 9.11 Hounsfield units, and clinically determined most likely stroke etiology was large artery atherosclerotic (LAA). Endovascular therapy was performed using a Sofia 6F (Plus) device. The patient was discharged with an NIHSS score of 10 and a 3-month follow-up modified Rankin Scale (mRS) score of 4. **(A)** Non-contrast computed tomography (NCCT). **(B)** CT angiography (CTA). **(C)** Overlay of NCCT and CTA. **(D)** Overlay of NCCT and CTA with segmentation of the thrombus in the basilar artery. Arrows indicate location of the thrombus. Shown are sagittal slices due to better visualization of the extent of the thrombus; actual thrombus segmentation was performed on axial slices.

Image analysis was performed as described previously (7). Briefly, NCCT and CTA scans were co-registered using a rigid transformation based on high-intensity signals. With the use of information from both NCCT and CTA, thrombi in the BA were segmented on consecutive axial slices. The proximal and distal ends of the thrombus were defined as (a) abrupt change of signal intensity in the CTA and/or (b) hyperdense signal in the NCCT. Thrombus material in the vertebral or P1 segment of the posterior cerebral arteries was not included in the segmentation (Figure 1). All segmentations were performed independently by AK and LS and averaged for further analyses. After confirming approximate normal distributions of voxel intensities within individual thrombi by visual inspection of intensity histograms, TP was calculated as mean thrombus signal intensity increase in the CTA relative to NCCT. Image analysis was done in ITK-SNAP v3.8 (8) and MRIcroGL (https://www.nitrc.org/projects/mricron).

TP was compared between groups using the Kruskal–Wallis test; proportions were compared using analysis of (co-)variance. We calculated multivariable binary logistic regression models including age, sex, and arterial hypertension [variables differed between cardioembolic and large artery atherosclerosis (LAA) groups in a univariate analysis; atrial fibrillation could not be included due to no cases of LAA patients] to test for a significant association between cardioembolic stroke etiology and outcome. Concordance between readers was quantified as intraclass correlation and determined using a two-way mixed model.

The study was designed and conducted in agreement with laws and regulations in the Federal State of Berlin according to which ethics committee approval and patient consent were not required for this retrospective study.

## Results

**Figure 2:**
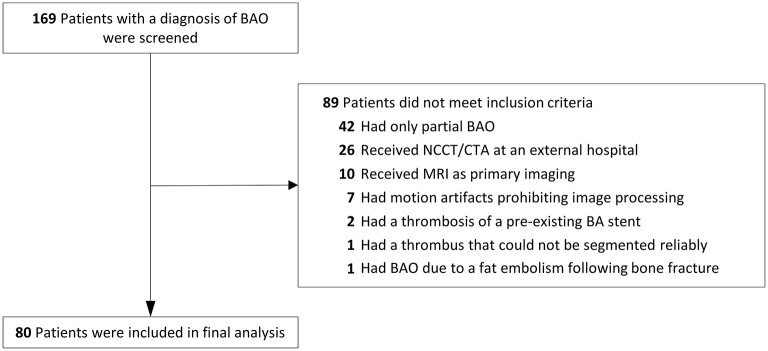
Flowchart. BA, basilar artery; BAO, basilar artery occlusion; NCCT, non-contrast computed tomography; CTA, computed tomography angiography; MRI, magnetic resonance imaging.

One hundred sixty-nine patients with a diagnosis of BAO were screened, of which 80 were included in the final analysis (Figure 2). Descriptive data of the patient population according to stroke etiology are presented in Table 1. Overall TAI values ranged from −20.8 to 29.1 Hounsfield units (HU); agreement between raters was excellent (intraclass correlation 0.97). Patients with cardioembolic stroke etiology (*N* = 36) were older and had higher rates of atrial fibrillation and arterial hypertension. Two patients had stroke of other determined etiology (one case was caused by dissection of the vertebral artery, and the other was due to a paraneoplastic clotting disorder that led to a paradoxical embolism and subsequent BAO). Intravenous thrombolysis with recombinant tissue plasminogen activator at a dose of 0.9 mg per kg body weight was administered in 50% of all patients. In all patients, endovascular clot removal was attempted, mostly with a Sofia 6F (Plus), a Sofia 5F, or a Trevo device.

**Table 1:** Baseline demographics and clinical parameters.

	**All patients** ** (*n* = 80)**	**Cardioembolic** ** (*n* = 36)**	**LAA** ** (*n* = 25)**	**Undetermined** ** (*n* = 17)**	**Other** ** (*n* = 2)**
Age, median (IQR)	72.8 (12)	75.9 (9.5)	72.5 (8)	69.7 (17.1)	46 (12.7)
Sex, % female (*n*)	42.5 (34)	47.2 (17)	40 (10)	41.2 (7)	0 (0)
**Cerebrovascular risk factors**					
Atrial fibrillation, % (*n*)	44.3 (35)	88.9 (32)	0 (0)	18.8 (3)	0 (0)
Diabetes, % (*n*)	11.5 (9)	2.9 (1)	24 (6)	12.5 (2)	0 (0)
Hypertension, % (*n*)	80 (20)	93.3 (14)	71.4 (5)	33.3 (1)	0 (0)
Hyperlipidemia, % (*n*)	35.4 (28)	33.3 (12)	44 (11)	31.3 (5)	0 (0)
Current smoker, % (*n*)	14.1 (10)	5.6 (2)	13 (3)	49 (4)	50 (1)
**Prior medication; antiplatelet or anticoagulants**					
None, % (*n*)	44.2 (34)	34.3 (12)	52.2 (12)	47.1 (8)	100 (2)
Aspirin only, % (*n*)	24.7 (19)	8.6 (3)	43.5 (10)	35.3 (6)	0 (0)
Dual antiplatelet therapy, % (*n*)	1.3 (1)	0 (0)	4.4 (1)	0 (0)	0 (0)
Vitamin K antagonist, % (*n*)	9 (7)	20 (7)	0 (0)	0 (0)	0 (0)
Oral anticoagulants, % (*n*)	19.5 (15)	34.3 (12)	0 (0)	17.7 (3)	0 (0)
NIHSS on admission, median (IQR)	20 (12–30)	18 (12–28)	24 (11–30)	26.5 (17–32)	12.5 (10–15)
rTPA, % (*n*)	50 (40)	27.8 (10)	76 (19)	56.3 (9)	100 (2)

*LAA, large artery atherosclerosis; IQR, interquartile range; NIHSS, National Institutes of Health Stroke Scale; rTPA, recombinant tissue plasminogen activator*.

**Figure 3:**
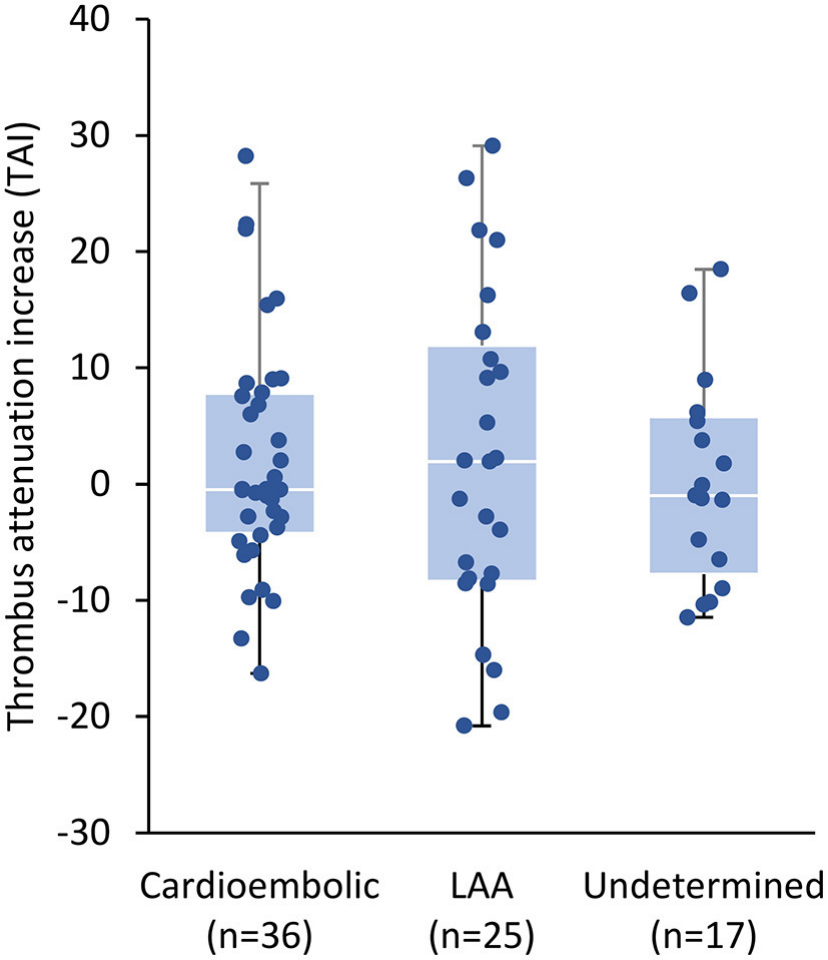
Boxplot of thrombus attenuation increase (TAI) assessed on baseline computed tomography imaging according to stroke etiology. The group “stroke of other determined etiology” only comprised two patients and is not shown in the chart. TAI did not vary significantly between groups (*p* = 0.955). The white lines within the boxes correspond to the distributions' median, the blue areas encompass values between the first and third quartiles, and the upper and lower whiskers extend to a maximum length of 1.5 times the interquartile range. LAA indicates large artery atherosclerosis.

There was no association between TP and stroke etiology: median (interquartile range [IQR]) values of TAI in patients with cardioembolic stroke, LAA stroke, stroke of undetermined origin, and stroke other determined etiology were −0.47 (−4.08 to 7.72), 1.94 (−8.14 to 10.75), −0.99 (−6.49 to 5.40), and −0.47 (−1.86 to 0.92), respectively (*p* = 0.955; Figure 3).

In a multivariable binary logistic regression model for the distinction of cardioembolic stroke from LAA stroke etiology, TAI was not independently associated with cardioembolic stroke etiology (odds ratio [OR] 1.0 [95 CI: 0.95–1.0], *p* = 0.751; Table 2).

**Table 2:** Binary logistic regression analysis for the distinction of cardioembolic stroke from large artery atherosclerosis stroke.

	**Univariate model**		**Multivariate model**	
	**Odds ratio (95% CI)**	***p*-value**	**Odds ratio (95% CI)**	***p*-value**
TAI, per unit increase	0.99 (0.96–1.04)	0.997	0.99 (0.95–1.04)	0.751
Age, per year increase	0.96 (0.90–1.02)	0.158	0.96 (0.89–1.04)	0.892
Male sex	1.34 (0.48–3.78)	0.577	1.05 (0.34–3.26)	0.928
Arterial hypertension	0.50 (0.12–2.09)	0.342	0.80 (0.15–4.29)	0.792

*TAI, thrombus attenuation increase*.

Mean thrombus density ranged from 22.1 to 74.7 HU (median 49.8, IQR = 41.6–55.8). There was no association between thrombus density and stroke etiology (*p* = 0.341). Median thrombus volume was 45.3 mm^3^ (IQR = 27.0–68.4) and did not differ between groups (*p* = 0.942).

Seventy-three patients were successfully recanalized by treatment. TP was nominally lower in recanalizers compared with non-recanalizers (median −0.49, IQR [−6.1 to 7.9] vs. 2.0 [−0.80 to 21.0]; *p* = 0.155). Following adjustment for age, sex, and stroke etiology, TP had a crude OR of 1.5 (95% CI: 0.89 to 2.6; *p* = 0.115) and adjusted OR of 1.39 (0.70–2.7), *p* = 0.345) for successful recanalization. In a subgroup of 24 patients with available 90-day mRS, TP had a crude and adjusted OR for favorable outcome (mRS ≤ 2) of 1.05 (95% CI: 0.97–1.1, *p* = 0.205) and 1.1 (95% CI: 0.94–1.2, *p* = 0.304).

## Discussion

In the current analysis, TP assessed on acute NCCT/CTA in patients with BAO was not associated with underlying stroke etiology.

Absolute values of TAI reported in this study were markedly lower than those reported previously in patients with BAO (mean 2.2 HU vs. ~18.9 HU) (9); this is most likely due to differing methodologies as described in detail previously (7). Briefly, here, an entire 3D thrombus segmentation across multiple consecutive slices was applied compared with previous reports in which spherical regions of interest (ROIs) were placed within thrombi on a best-fitting axial slice and negative values were set to zero (6, 9, 10). Despite the discrepancy of absolute values reported, we also observed higher TAI measurements in patients with LAA stroke compared with non-LAA stroke, although this difference was not statistically significant.

Interestingly, previous studies found that high TAI values in patients with M1 occlusion were associated with cardioembolic stroke etiology (6, 7). Therefore, one might have expected a similar observation in BAO patients; however, this was not the case. In contrast to the MCA, the BA can be perfused anterograde as well as retrograde in the case of occlusion, depending on the local perfusion pressures. Furthermore, a much higher rate of LAA BAO occurs due to local thrombosis of underlying stenosis, whereas M1 occlusions are more often caused by embolism from proximal arteries (11). These pathophysiological differences likely contribute to altered clot characteristics and may affect TAI depending on occlusion site and characteristics of the circle of Willis. Also, the close proximity of the BA to the skull base might affect perviousness measurements. However, a comprehensive analysis on effect of thrombus location on TP is still lacking.

It was recently suggested that TP might differentiate between BAO with and without underlying basilar stenosis (9). In our study, information on basilar stenosis as assessed on digital subtraction angiography was not available. Instead, we examined the relationship between TP and stroke etiology defined by TOAST criteria and found no significant association, neither in a univariable analysis nor in a multivariable analysis. These results are in line with findings from a recent study, which—in contrast to patients with M1 occlusions (6)—found no association between histological thrombus composition and stroke etiology in patients with BAO (12). The available evidence therefore suggests that the potential role of TP measurements to guide determination of stroke etiology might be limited to occlusions in the anterior circulation.

The primary limitations of this study include its retrospective nature and relatively small sample size, which increase the likelihood of type II errors. Nonetheless, considering BAO makes up only ~2% of all acute ischemic strokes (2), we believe that these results of a consecutive cohort of 80 BAO patients are valuable to further our understanding of the diagnostic value of TP as a novel imaging marker. Secondly, there was no central assessment of stroke etiology; rather, stroke cause was determined by the treating physicians on certified stroke units at the admitting hospital. Thirdly, we did not perform histological analyses of the retrieved thrombi and therefore could not assess any relationship between thrombus composition and TP measures or outcome. Lastly, clinical follow-up was only available for a subset of patients.

## Conclusion

In conclusion, we found no association between TP assessed on acute NCCT/CTA in patients with BAO and stroke etiology. Additional studies are required to identify the mechanisms by which thrombus location affects the relationship between TP and stroke etiology. In the meantime, confirmatory studies to establish the potential role of TP for clinical applications should focus on patients with anterior circulation stroke.

## Data Availability Statement

The data that support the findings of this study are available in anonymized form from the corresponding author upon reasonable request. The data are not publicly available due to their containing information that could compromise the privacy of research participants.

## Ethics Statement

Ethical review and approval was not required for this retrospective study on human participants in accordance with the local legislation and institutional requirements. Written informed consent for participation was not required for this study in accordance with the national legislation and the institutional requirements.

## Author Contributions

LS, AK, and CN conceived and designed the study. LS developed the pipeline for image analysis. AK and LS performed thrombus segmentations and prepared the manuscript. AK performed the statistical analyses. All authors contributed to acquisition, analysis of the data and critical revision of the manuscript, and figures for intellectual content.

## Funding

LS and AK are participants in the Berlin Institute of Health-Charité (Junior) Clinical Scientist Program funded by the Charité—Universitätsmedizin Berlin and the Berlin Institute of Health. ME received funding from Deutsche Forschungsgemeinschaft (DFG, German Research Foundation) under Germany's Excellence Strategy—EXC-2049-390688087 and from Bundesministerium für Bildung und Forschung (BMBF; German Ministry for Education and Research) for the Center for Stroke Research Berlin. CL is a participant in the Berlin Institute of Health-Charité Clinical Fellow Program funded by the Charité—Universitätsmedizin Berlin and the Berlin Institute of Health.

## Conflict of Interest

ME reports grants from Bayer and fees paid to the Charité from Bayer, Boehringer Ingelheim, BMS, Daiichi Sankyo, Amgen, Sanofi, Novartis, and Pfizer, all outside the submitted work. MS reports no conflicts of interest related to this work. He is a patent holder and shareholder of PhantomX a company producing 3D models for CT. CL reports personal fees from Edwards Lifesciences and fees paid to Charité from Bard, Zoll, and Pfizer, all outside the submitted work. CN reports personal fees from Boehringer Ingelheim, Pfizer Pharma, Britsol-Myers Squibb, Abbott, and Portola, outside the submitted work. LS reports personal fees from Daiichi Sankyo, outside the submitted work. The remaining author declares that the research was conducted in the absence of any commercial or financial relationships that could be construed as a potential conflict of interest.

## Publisher's Note

All claims expressed in this article are solely those of the authors and do not necessarily represent those of their affiliated organizations, or those of the publisher, the editors and the reviewers. Any product that may be evaluated in this article, or claim that may be made by its manufacturer, is not guaranteed or endorsed by the publisher.
